# Membrane Cholesterol Removal Changes Mechanical Properties of Cells and Induces Secretion of a Specific Pool of Lysosomes

**DOI:** 10.1371/journal.pone.0082988

**Published:** 2013-12-20

**Authors:** Barbara Hissa, Bruno Pontes, Paula Magda S. Roma, Ana Paula Alves, Carolina D. Rocha, Thalita M. Valverde, Pedro Henrique N. Aguiar, Fernando P. Almeida, Allan J. Guimarães, Cristina Guatimosim, Aristóbolo M. Silva, Maria C. Fernandes, Norma W. Andrews, Nathan B. Viana, Oscar N. Mesquita, Ubirajara Agero, Luciana O. Andrade

**Affiliations:** 1 Departamento de Morfologia, Instituto de Ciências Biológicas, Universidade Federal de Minas Gerais, Belo Horizonte, MG, Brazil; 2 LPO-COPEA, Instituto de Ciências Biomédicas, Universidade Federal do Rio de Janeiro, Rio de Janeiro, RJ, Brazil; 3 Departamento de Física, Instituto de Ciências Exatas, Universidade Federal de Minas Gerais, Belo Horizonte, MG, Brazil; 4 Departamento de Bioquímica e Imunologia, Instituto de Ciências Biológicas, Universidade Federal de Minas Gerais, Belo Horizonte, MG, Brazil; 5 Instituto de Microbiologia Paulo de Góes, Universidade Federal do Rio de Janeiro, Rio de Janeiro, RJ, Brazil; 6 Departamento de Microbiologia e Parasitologia, Instituto Biomédico, Universidade Federal Fluminense, Rio de Janeiro, RJ, Brazil; 7 Department of Cell Biology and Molecular Genetics, University of Maryland, College Park, Maryland, United States of America; 8 Instituto de Física, Universidade Federal do Rio de Janeiro, Rio de Janeiro, RJ, Brazil; Cambridge University, United Kingdom

## Abstract

In a previous study we had shown that membrane cholesterol removal induced unregulated lysosomal exocytosis events leading to the depletion of lysosomes located at cell periphery. However, the mechanism by which cholesterol triggered these exocytic events had not been uncovered. In this study we investigated the importance of cholesterol in controlling mechanical properties of cells and its connection with lysosomal exocytosis. Tether extraction with optical tweezers and defocusing microscopy were used to assess cell dynamics in mouse fibroblasts. These assays showed that bending modulus and surface tension increased when cholesterol was extracted from fibroblasts plasma membrane upon incubation with MβCD, and that the membrane-cytoskeleton relaxation time increased at the beginning of MβCD treatment and decreased at the end. We also showed for the first time that the amplitude of membrane-cytoskeleton fluctuation decreased during cholesterol sequestration, showing that these cells become stiffer. These changes in membrane dynamics involved not only rearrangement of the actin cytoskeleton, but also *de novo* actin polymerization and stress fiber formation through Rho activation. We found that these mechanical changes observed after cholesterol sequestration were involved in triggering lysosomal exocytosis. Exocytosis occurred even in the absence of the lysosomal calcium sensor synaptotagmin VII, and was associated with actin polymerization induced by MβCD. Notably, exocytosis triggered by cholesterol removal led to the secretion of a unique population of lysosomes, different from the pool mobilized by actin depolymerizing drugs such as Latrunculin-A. These data support the existence of at least two different pools of lysosomes with different exocytosis dynamics, one of which is directly mobilized for plasma membrane fusion after cholesterol removal.

## Introduction

Cholesterol-enriched membrane microdomains, known as membrane rafts, are platforms containing specific proteins and lipids that are responsible for coordinating several cellular processes. Membrane rafts have been proposed to regulate several cellular events such as intracellular signaling cascades [1], [2], [3], [4], cellular migration [5], interactions between plasma membrane and cytoskeleton through lipid (e.g PIP2) and protein components (e.g Rho-GTPases, integrins) [6], membrane trafficking [7] and vesicle exocytosis [8], [9].

Although cholesterol-enriched microdomains regulate many cellular processes we have particularly focused our attention in their role in lysosomal exocytosis. Lysosomes are acidic organelles that participate not only in intracellular degradation but also in other cellular events, including plasma membrane repair after injury [10]. In the latter, lysosomal exocytosis was shown to release acid sphingomylinase (ASM), an enzyme that cleaves sphingomyelin in the outer leaflet of the plasma membrane generating ceramide, which in turn induces a compensatory form of endocytosis responsible for repairing the injured membrane [11]. Exocytosis of lysosomes at plasma membrane injury sites is regulated by synaptotagmin VII, a calcium sensor protein present in these organelles [12]. We and others have shown that cholesterol removal can cause lysosomal exocytosis in fibroblasts [13], epithelial cells [14] and cardiomyocytes [15]. Exocytic events induced by cholesterol sequestration have also been described in other cellular models, such as neurons. Sequestration of cholesterol from crayfish motor nerve terminals or hippocampal neurons in culture led to an increase in spontaneous exocytosis of synaptic vesicles [8], [9] in a calcium independent manner. In this model, a reduction in evoked exocytosis was also reported [9], [16]. However, despite the extensive evidence for exocytosis induced by cholesterol removal, there is still no well-defined mechanism to explain this phenomenon.

Cholesterol-containing membrane microdomains have been described to interact with the cytoskeleton [6], and a proteomic approach showed co-localization between cytoskeleton-binding proteins and raft regions [17]. Since then, a series of other studies described the impact of raft disruption by cholesterol extraction on the organization of the actin cytoskeleton and its influence on cellular structure. In 2003, Kwik and coworkers showed that removal of cholesterol from fibroblast membranes caused a reduction in the mobility of some transmembrane proteins, due to reorganization of the cytoskeleton [18]. Later, it was demonstrated that cholesterol sequestration from endothelial cells led to an increase in both cellular rigidity [19] and in the attachment between plasma membrane and cytoskeleton. Simultaneously, a decrease in lipid diffusion coefficient was also observed [20]. Additionally, in 2009 Qi and collaborators demonstrated that cholesterol sequestration, in immortalized osteoblasts, led to stress fiber formation via Rho activation [21]. Taken together, these studies revealed the importance of cholesterol in regulating the dynamics of cytoskeleton-mediated processes.

In the present work, we investigated whether changes in membrane-cytoskeleton dynamics and cellular mechanical properties could be correlated with lysosomal secretion. Our results demonstrated that cholesterol removal led to actin polymerization and modification of mechanical properties of cells, including surface tension and bending modulus. Additionally, using defocusing microscopy technique, we showed a change in the relaxation time and amplitude curvature, confirming that cells became more rigid during the cholesterol extraction treatment. We also showed that these changes in the actin cytoskeleton and membrane dynamics were correlated with the secretion of a specific pool of lysosomes.

## Materials and Methods

### Ethics Statement

All animals were maintained in our animal facilities in compliance with the guidelines of the UFMG (Universidade Federal de Minas Gerais) ethics committee for the use of laboratory animals (protocol 45/2009 approved by CETEA-UFMG) and are in accordance with CONCEA, the Brazilian institution that regulates animal husbandry.

### Cell Cultures

Most of the experiments were performed using a cloned mouse fibroblast cell line (WTCL3). These fibroblasts were derived from wild type (WT) C57BL/6J embryos and generated in Dr. Paul Saftig’s laboratory (University of Kiel, Germany) by spontaneous immortalization of primary embryonic fibroblasts in culture, around passages 10–20 [22]. Cells were maintained in culture by consecutive passages in high glucose DMEM (Invitrogen, Carlsbad, CA, USA) supplemented with 10% FBS, 1% penicillin-streptomycin and 1% glutamine, as described previously [23]. For siRNA assays we used NRK (*Natural Rat Kidney*) cells, which were also maintained in DMEM supplemented with 10% FBS, 1% penicillin-streptomycin and 1% glutamine. Primary cardiomyocyte cultures, used in the lysosomal exocytosis assay in the presence of BAPTA-AM, were prepared as described previously [15]. Briefly, 15 newborn mice were killed by decaptation and had their hearts removed aseptically. Those hearts were minced and submitted to trypsin and collagenase digestion. In the end of isolation procedure, enzymes were inactivated and cells were plated in 24 well plates at a density of 1,0×10^5^ cells/well. Experiments were performed after 72 hours of culture.

For all experiments, except for primary cardiomyocyte cultures, cells were plated 24 hours in advance. For lysosomal exocytosis assays or actin labeling, the fibroblasts were plated on 24 well plates containing 13 mm round coverslips at a density of 5.0×10^4^ cells/well. For tether extraction or defocusing microscopy experiments, fibroblasts were plated on 35 mm glass-bottom dishes at a density of 2.0×10^5^ cells/well. For cholesterol quantification and Rho activation assays fibroblasts were plated on 6 well plates at a density of 5.0×10^4^ cells/well or on 60 mm culture dishes at a density of 2.5×10 ^6^ cells/well, respectively. For knockdown experiments, NRK cells were seeded on 6 well plates at a density of 1.25×10^5^ cells/well.

### Drug Treatments

All reagents used were acquired from Sigma-Aldrich (St. Louis, MO, USA) unless otherwise stated. For plasma membrane cholesterol sequestration, cells were first washed three times with Hank’s Balanced Salt Solution supplemented with 1.8 mM of CaCl_2_.2H_2_O (HBSS +), and then incubated with 5 or 10 mM methyl-beta cyclodextrin (MβCD) solutions diluted in DMEM high glucose (Invitrogen, Carlsbad, CA, USA), in the absence of serum, for 45 minutes at 37°C and 5% CO_2_. After treatment, cells were washed three times with HBSS+ and subjected to experimental assays. For F-actin cytoskeleton disruption assays, fibroblasts were incubated with 95 nM Latrunculin-A (Lat-A) for 1 hour at 37°C and 5% CO_2_, washed three times with HBSS+ and incubated with DMEM alone (without serum) or in the presence of 5 or 10 mM of MβCD, for 45 minutes.

### Quantification of Plasma Membrane Cholesterol

In order to quantify the amount of cholesterol in control and MβCD treated cells, fibroblasts were first washed with HBSS+ and then incubated with phosphate buffered saline containing 0.02 mM of ethylenediamine tetraacetic acid (PBS- EDTA) for 5 minutes at 37°C and 5% CO_2_. The detached fibroblasts were collected, counted using a Neubauer chamber and submitted to lysis for 1.5 h in a 4°C ice-cold buffer solution containing 100 mM Tris-HCl (pH 8.0), 150 mM NaCl, 2 mM MgCl_2_, 1% Triton X-100, 5 mM iodoacetamide, 0.025% NaN_3_, 1 mM PMSF, 1 mM di-isopropylfluorophosphate and 0.02 U/mL of aprotinin, with gentle stirring. After lysis, small aliquots of the cellular extracts were collected for protein quantification using the Bradford method (Bio-rad, Brazil) [24]. The remaining lysates were submitted to the Folch lipid extraction method [25]. Briefly, the extracts were diluted in a 8∶4:3 chloroform:methanol:sample ratio, the upper aqueous phase was removed and the organic phase was dried under N_2_. Dried non-polar lipids were subjected to cholesterol quantification using an Amplex Red Cholesterol Assay Kit (Invitrogen, Carlsbad, CA) according to the manufacturer’s instructions. Untreated and cholesterol-sequestered fibroblasts apolar lipids were suspended in 500 µL of methanol and 5 µL of these solutions were diluted in 45 µL of the reaction buffer (1∶10) and added to a 96 well black microplate. After that, 50 µL of a solution containing Amplex Red Reagent/Horseradish Peroxidase (HRP)/cholesterol oxidase/cholesterol esterase were added to every well and the reaction was incubated for 30 minutes, at 37°C, protected from light. The reaction buffer (without cholesterol) alone or containing 10 mM of H_2_O_2_ were used as negative and positive controls, respectively. The fluorescence levels were measured in a fluorimeter (Gemini XPS- Molecular Devices, Sunnyvale, CA, USA) using of 560 nm for excitation and 590 nm for emission. The amount of cholesterol was quantified for all the experimental conditions.

### Labeling of Polymerized Actin

After drug treatment, fibroblasts were washed 3 times with HBSS+ and fixed with paraformaldehyde (PFA) 4% in PBS for 15 minutes at room temperature. Cells were rinsed 3 times with PBS and permeabilized with Triton X-100 0.02% for 5 minutes before incubation with PBS/BSA 5% for 30 minutes. In sequence, cells were incubated with 50 µg/mL of Phalloidin-FITC in PBS for 2 hours. Following, monolayers were washed 3 times with PBS, incubated with 4′,6-Diamidino-2-Phenylindole, Dihydrochloride (DAPI) for 5 minutes, washed 4 times with PBS and mounted in glass slides using FluorSave™ Reagent (Calbiochem-Millipore, Billerica, MA, USA). Images were captured using a TCS-SP5 II confocal microscope (Leica Microsystems, Germany) equipped with LAS AF 2.2.0 Software (Leica Microsystems, Germany).

### Rho-GTP Pull-down Assay

In order to quantify Rho-GTP activation upon MβCD treatment, fibroblasts were incubated with MβCD 10 mM for 20 or 45 minutes. After incubation, cells were washed with ice-cold TBS (25 mM Tris- HCl, pH 7.5 and 150 mM NaCl) and lysed using the lysis buffer provided by Active Rho Pull-Down and Detection Kit (Thermo Scientific Waltham, MA, USA), together with a protease inhibitor cocktail (Aprotinin 0.8 µM; 2 mM PMSF, 0.01 mM Pepstatin-A, 20 µM Leupeptin and 5 mM EDTA pH 8.0). Cell extracts were then collected and centrifuged at 16.000 g for 15 minutes and used for the pull down assay according to the manufacturer’s instructions, as follows. As an internal control, a small fraction of each cellular extract (20 µL of each group) was collected in order to measure the total amount of Rho present in each sample. The extracts were transferred to collection tubes coupled to spin cups, each tube containing 100 µL of glutathione resin slurry and 80 µL of GST-Rhotekin-RBD. Cell extracts for incubation with GDP were also prepared as negative controls for the pull-down assay. After transference, the tubes were incubated at 4°C for one hour, centrifuged at 6.000 g for 30 seconds and washed with lysis/binding/washing buffer. Then the eluates and cell extracts received 50 µL of sample buffer SDS 2X containing β-mercaptoethanol (provided by the kit) and were boiled for 5 minutes. The samples (together with a molecular marker, Bio-rad Brazil) were run on a 10% SDS-PAGE acrylamide gel and transferred to a PVDF membrane (Millipore, Billerica, Massachusetts, USA). The membrane was blocked with TBS, supplemented with 3% of bovine serum albumin (BSA), for 2 hours, at room temperature and incubated, at 4°C, with 10 mL of TBST with 3% of BSA and 15 µL anti-Rho primary antibody overnight. The membrane was then washed 5 times with TBST and incubated for one hour at room temperature with horseradish peroxidase-conjugated anti-rabbit antibody (1∶2,000 diluted in TBST 5% non-fat dry milk). After washes, immunoreactive GTP-bound Rho was detected by enhanced chemiluminescence (ECL) according to the manufacturer’s instructions (GE Healthcare, Pittsburgh, PA, USA). Densitometric analysis was performed with ImageJ software (National Institutes of Health, Bethesda, MD, USA).

### Membrane Tether Extraction Experiments with Optical Tweezers

Fibroblasts were treated with 5 or 10 mM MβCD, washed 3 times with HBSS+, replenished with fresh media, and submitted to tether extraction using optical tweezers (OT). The OT system used employed an infrared Nd:YVO_4_ Osprey laser with a wavelength of 1.064 µm (Quantronix, East Setauket, NY, USA). The laser has a Gaussian intensity profile (TEM_00_ mode) and a beam half width of 2.3±0.2 mm at the back focal plane of the objective lens. The infrared laser is coupled to an Eclipse TE300 inverted microscope (Nikon, Melville, NY, USA) equipped with a PLAN APO 100X 1.4 N.A. DIC H objective that generates the optical trap. The OT calibration and tether extraction experiments were performed as described previously [26], [27]. Briefly, WTCL3 fibroblasts treated with 5 or 10 mM MβCD received a 1 µL solution of uncoated polystyrene beads of 1.52±0.2 µm radius (Polysciences Inc, Warrington, PA, USA) and each dish was placed on the microscope. One bead was captured with the OT and pressed against the membrane of a chosen cell for 5 seconds, in order to attach to it. The motorized microscope stage (Prior Scientific, Rockland, MA, USA) was set to move with a controlled velocity of 1 µm/s. Images of the entire process were collected by a charge-couple device (CCD) C2400 camera (Hamamatsu, Japan) and digitalized by a Scion FG7 frame grabber (Scion, Torrance, CA, USA) using a frame rate of 10 frames/second. Variations in the trapped bead equilibrium position as a function of the stage displacement was determined using ImageJ software (National Institutes of Health, Bethesda, MD, USA). Using the trap calibration, the displacement of the bead’s center of mass was converted to measured force. All the experiments were performed in an environmental chamber that maintained 37°C temperature and 5% CO_2_. The data were analyzed using Kaleidagraph software (Synergy Software, Essex Junction, VT, USA).

### Membrane Tether Radius Measurements by Scanning Electron Microscopy (SEM)

In order to measure the tether radius extracted from control and MβCD treated cells, immediately after extracting the tethers, cells were fixed using a solution containing 2.5% glutaraldehyde in 0.1 M cacodylate buffer (pH 7.4) for 1 hour, at room temperature. Fixed fibroblasts were rinsed with 0.1 M cacodylate buffer and incubated with OsO_4_ 1% in cacodylate buffer 0.1 M, for 40 minutes. Cells were then dehydrated using an ethanol series ranging from 10 to 100%. After dehydration, samples were removed from culture dishes, dried at a CPD 030 Critical Point Drier (BAL-TEC, Liechtenstein) and mounted on specimen stubs. The next step was to metalize the samples using a SCD 050 Sputter Coater (BAL-TEC, Liechtenstein) and observe the tethers in a FEI QUANTA 250 scanning electron microscope (FEI Company, Hillsboro, OR, USA). In order to measure the tether radius, we used the methodology previously established [27]. After measuring the tether radius, *R*, and the tether force, *F_0_*, both cell surface tension, *σ*, and bending modulus, *κ*, were determined using the following equations:
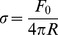
(1)

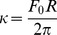
(2)


### Defocusing Microscopy

Defocusing microscopy (DM) technique was used to analyze structural modifications in both plasma membrane and cytoskeleton of fibroblasts during treatment with 5 or 10 mM of MβCD, in comparison to control untreated cells. With this method, it is possible to turn a phase-transparent object (like adhered cells) into a visible object by introducing defocalization in the microscope [28], [29]. We recorded control fibroblasts by DM in serum free DMEM High Glucose for 10 minutes, then added 5 or 10 mM of MβCD to the medium and continued recording this cell for another 45 minutes. All experiments were analyzed using ImageJ *Plugins* that correct for background and calculate the temporal autocorrelation function among frames. These temporal autocorrelation functions were adjusted in KaleidaGraph Software (Synergy Software, Essex Junction, VT, USA) using single exponential decay curves that carry information about their amplitude and time characteristics. The experiments were performed in a Nikon Eclipse TI inverted microscope equipped with a 530 nm wavelength green filter, a stage-heated oil-immersion objective Nikon Apo Tirf 100X, NA 1.49 (Nikon, Japan), and a an environmental chamber (model Chamlide IC- CU:109, Live Cell Instrument, Nowan-gu, Korea) which provides a 5% CO_2_ atmosphere, 37°C temperature and 50% humidity. The images were captured using a 12 bit Uniq camera (model 1800 CL) (Epix Inc, Buffalo Grove, IL, USA, 4096 gray levels and 0.064 µm of pixel square side), with a gain of 11.04 db and a capture rate of 1 frame per second. The focal distance was controlled during the entire experiment by a Nikon Perfect Focus System (PFS) apparatus and the camera gray level calibration was performed as previously described [28].

### Lysosomal Exocytosis Assay

In order to quantify the amount of lysosomal exocytosis upon cholesterol removal and/or cytoskeleton disruption by treatment with MβCD or Lat-A, respectively, a time and dose-dependent β-hexosaminidase (β-hex) secretion assay was performed as previously described [15]. Briefly, cells previously treated or not with Lat-A were incubated with 5 or 10 mM of MβCD for 10, 20 or 40 minutes. Untreated cells and ionomycin treated cells (10 µM, 10 minutes) were used, respectively, as negative and positive controls. For the exocytosis assays in primary cardiomyocyte cultures, cells were treated with 10 mM of MβCD in the presence or absence of 1 mM of BAPTA-AM. After treatment, 350 µL of the cell supernatant media were collected and the cells were lysed using 1% Triton X-100 in PBS. The recovered media and 50 µL of the cellular lysates were incubated with 6 mM of β-hex substrate (4-methylumbelliferyl-N-acetyl-B-D-glucosaminide) dissolved in sodium citrate-phosphate buffer, pH 4.5. The reactions were stopped by adding to each sample 100 µL of the stop solution (2 M Na_2_CO_3_.H_2_O and 1.1 M glycin) followed by reading in a spectrofluorimeter at 365 nm excitation and 450 nm emission (Synergy 2, Biotek, Winooski, VT, USA). The results were expressed as secreted β-hex as a percentage of the total enzyme content in the cells.

### Lysosomal Dispersion Analysis

Lysosomal dispersion analysis was performed as described previously [15]. Briefly, control fibroblasts and fibroblasts treated with either MβCD alone or after Lat-A were fixed and immunostained with anti-LAMP-1 and DAPI as described previously [15]. Images were taken using a 100× objective with N.A. of 1.30 at Olympus BX51 fluorescence microscope (Shinjuku, Tokyo, Japan). To each image (associated to a specific drug treatment), we computed the mean center position and the mean radius (R) of each nucleus. A computational code has been written using the IDL (Interactive Data Language) programming language in order to calculate the lysosomal dispersion distribution within the cells from the different experimental groups. Subsequently, an average distance (D) was computed by using the distances of each lysosome relative to the cell center. Lysosomes farther than 1.5 radii from cell center were excluded from the computation of this average value since the majority of lysosomes were found nearer than 1.5 in relation to the cell nuclei. Moreover, when increasing D values, lysosomes from neighboring cells could be taken into account in the dispersion calculation. Finally, the mean lysosome distance (D) relative to the mean nucleus’ radius (R) was defined as the ratio D/R. Such procedure was repeated for the maximum number of isolated cells available from the image sets of each drug treatment. The results of this analysis are a distribution of D/R values associated to each drug treatment, and are represented as histograms.

### siRNA Silencing of Synaptotagmin VII

In order to investigate the role of synaptotagmin VII (Syt-VII) in lysosomal exocytosis triggered by cholesterol removal, we performed siRNA knockdown experiments with NRK cells. Syt-VII knockdown was performed using Lipofectamine RNAiMAX Reagent (Invitrogen, Carlsbad, CA, USA), according to manufacturer’s instruction with the syt-VII siRNA oligo (Invitrogen, Carlsbad, CA, USA, Syt7RSS339874 5′-GCCAACUCCAUCAUCGUGAACAUCA-3′). As a control, a medium GC siRNA oligo (random oligo) (Invitrogen, Carlsbad, CA, USA) was used in parallel. After transfection, cells were harvested either for lysosomal exocytosis experiments, as described above, or used for RT-PCR to assess level of Syt-VII silencing.

For RT-PCR, total RNA was isolated using Trizol reagent (Invitrogen, Carlsbad, CA) according to manufacturer’s instructions. cDNA was prepared from total RNA using the SuperScript III kit (Invitrogen, Carlsbad, CA, USA) in accordance with the company’s instructions. The RT-PCR reaction was performed to verify the presence or the absence of syt-VII cDNA using the following primers: forward 5′-GGG TTT CCC TAT GAG AAA GTG GT-3′ and reverse 5′-CCT TCC AGA AGG TCT GCA TCT GG-3′. Alternatively, as an experimental control, we also amplified actin, a housekeeping gene, using the following primers: forward 5′-CCG TAA AGA CCT CTA TGC CA-3′ and reverse 5′-CAT CTG CTG GAA GGT GGA CA-3′. The PCR reaction was performed with a final volume of 10 µL containing *Taq* DNA Polymerase buffer (Tris-HCL 10 mM pH 8.4, KCl 50 mM, 0,1% Triton X-100, MgCl2 1.5 mM), 2 pmol of each specific primer, 2,5 units of Taq DNA Polymerase (Phoneutria, Brazil) and 1 µL of cDNA. The mixture was submitted to 35 cycles of 1 minute at 95°C for denaturation, 1 minute at 55°C for annealing and 1 minute at 72°C for extension in an Eppendorf, termocycler (Hamburg, Germany). In the last cycle extension was performed for 10 minutes. PCR products were submitted to electrophoresis in agarose gel 0,1% (wt/vol) and stained with ethidium bromide. The intensities of the bands on the agarose gel were measured using ImageJ software (National Institutes of Health, Bethesda, MD, USA).

### Statistical Analysis

All data were represented as mean ± standard error. Student t-test or One-way ANOVA followed by Neuman-Keuls post hoc comparison test were performed in order to calculate statistical differences between control and treated groups. All statistical analyses were performed using GraphPad Prism 4 software (La Jolla, CA, USA). For histogram distributions, the cumulative frequency was calculated and analyzed using the Kolmogorov-Smirnov statistical test, performed using IDL. Values of p<0.05 were considered statistically significant.

## Results

### Fibroblasts Treated with MβCD had their Cholesterol Content Efficiently Sequestered

In order to evaluate the effectiveness of MβCD treatment in sequestering cellular cholesterol from immortalized fibroblasts, we performed a quantitative assay to measure the amount of this lipid in control (non-treated) and in cells treated with 5 or 10 mM MβCD, using the Amplex Red Cholesterol Assay Kit (Invitrogen, Carlsbad, CA, USA). This quantitative assay confirmed that cholesterol was efficiently sequestered from fibroblasts treated with either 5 or 10 mM of MβCD. Cells incubated with 5 mM MβCD presented 4.5 times less cholesterol whereas cells treated with 10 mM of MβCD showed 6.2 times less cholesterol, when compared to control fibroblasts (Table 1).

**Table 1:** Incubation with MβCD diminished cholesterol content in fibroblasts.

	Cholesterol content (mmol/mL)	Total protein extract (mg/mL)	Cholesterol/protein (mmol/mg)
Control	9,102±0.001	0,247±0.001	0,037±0.001
MβCD 5 mM	2,818±0.001	0,344±0.001	0,008±0.001
MβCD 10 mM	2,417±0.001	0,407±0.001	0,006±0.001

The first column shows the values obtained by cholesterol quantification in the fluorimeter, the second column enumerates the values of total protein amounts obtained by Bradford method and the third column indicates the amount of cholesterol normalized by the total amount of protein in each experimental group.

### Cholesterol Removal Alters Organization of the Actin Cytoskeleton via Rho Activation

**Figure 1:**
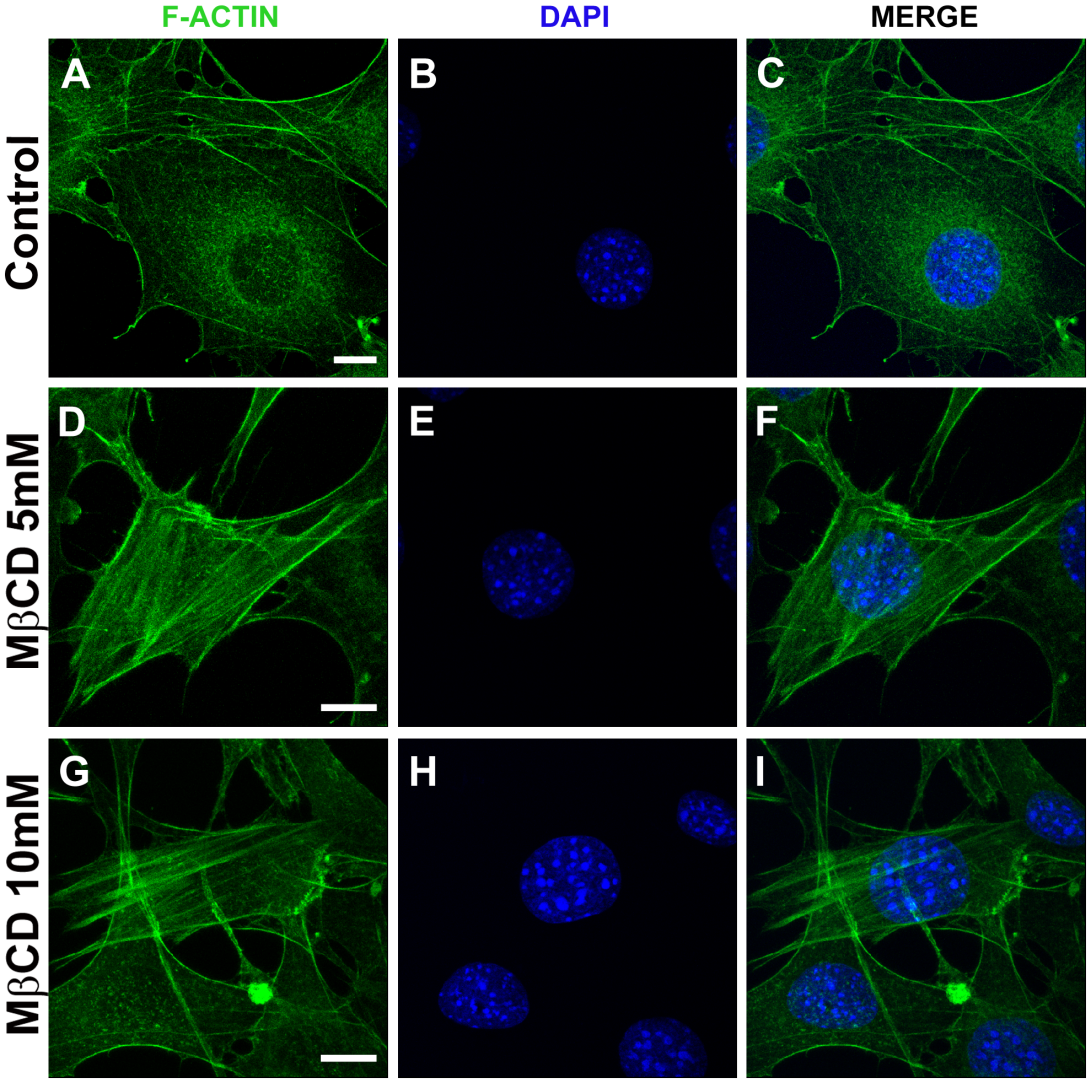
Cholesterol removal changed actin cytoskeleton organization. Representative images of actin cytoskeleton from fibroblasts untreated (A–C) or treated with either 5 (D–F) or 10 (G–I) mM of MβCD for 45 minutes at 37°C, fixed and labeled with Phalloidin-FITC and DAPI (cell nuclei). Three independent experiments were performed in triplicates and at least 50 different cells were observed per group. Each selected image represents the most predominant actin cytoskeleton cell morphology of that experimental condition. Scale bars are all 10 µm.

**Figure 2:**
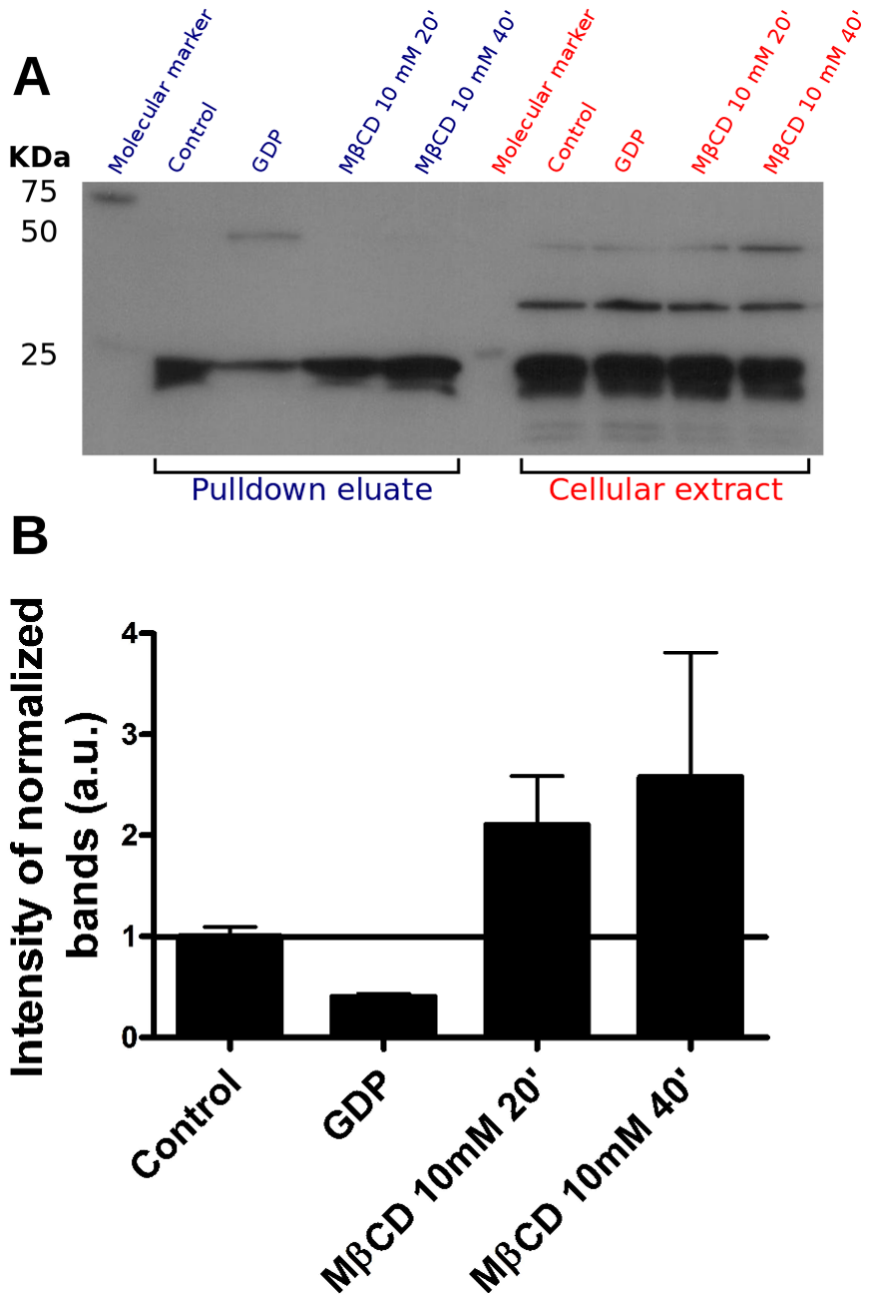
Cholesterol sequestration was able to activate Rho upon treatment with MβCD. Fibroblasts, treated with MβCD 10 mM, for 20 or 40 minutes, were submitted to Rho-GTP pull down assay. In (A), left side, we have the western-blotting result of the pull-down eluates showing that after 20 or 40 minutes of cholesterol removal Rho is activated in fibroblasts. In (A), right side, we have cell lysates that were not submitted to the pull-down assay showing the total amount of Rho present in each experimental group. Control untreated fibroblasts and cell extract incubated with GDP were used as controls of the Rho activation and of the pull-down technique, respectively. (B) Graph showing the densitometric analysis of activated Rho seen in the autoradiogram of the upper panel (A) normalized by the corresponding band at the cell lysates. The data are mean ± standard error of two independent experiments. Data are expressed in arbitrary units (folds in relation to control).

Proteomic studies have demonstrated that many cytoskeleton-binding proteins are located in plasma membrane cholesterol-enriched platforms [17], [30]. These findings, together with additional data from the literature, have demonstrated the importance of cholesterol-enriched microdomains in establishing connections between the plasma membrane and the cytoskeleton (reviewed by Chichili and Rodgers, 2009 [31]). Therefore, we decided to examine the organization of the actin cytoskeleton upon cholesterol removal from the plasma membrane in our fibroblast cultures. Figure 1A–C, shows the profile of filamentous actin (F-actin) distribution in control fibroblasts. F-actin was visualized homogenously distributed throughout the entire cell. In contrast, when cells were treated with 5 (Fig. 1D–F) or 10 mM (Fig. 1G–I) MβCD, there was F-actin redistribution with more evident stress fibers when compared to untreated fibroblasts. These results revealed that cholesterol removal from plasma membrane changes the actin cytoskeleton organization in fibroblasts, confirming previous results from Qi and co workers (2009) [21]. These authors also demonstrated that the actin cytoskeleton reorganization and stress fiber formation occurred via Rho activation. In order to verify whether the effect on actin observed in our system could also be a consequence of Rho activation, we performed a Rho-GTP pull-down assay. Eluates obtained from fibroblasts cell lysates after the pull-down assay (Fig. 2A, left side), treated or not with MβCD, were run on a gel and transferred to a PVDF membrane and detected with an Anti-Rho GTP antibody. Simultaneously, cell extracts (containing the total amount of Rho) were also run on the gel and transferred to a PVDF membrane (Fig. 2A, right side). These inputs were used to normalize the values, obtained by the densitometry analysis, for the eluates. We observed that after 40 minutes of exposure to MβCD there was an almost 2 fold increase in the amount of activated Rho (Rho-GTP) when compared to control untreated cells (Fig. 2A and B). Rho activation was also measured in cell extracts incubated with GDP, as a negative control (Fig. 2A). Figure 2A also shows the cellular extracts, indicating that the amount of protein loaded in each lane was similar. In agreement with and also reinforcing previous results, we showed that cholesterol sequestration, due to MβCD treatment, induces Rho activation, which is probably responsible for the observed actin reorganization pattern.

### Cholesterol Removal Induces “*de novo*” Formation of Actin Filaments

**Figure 3:**
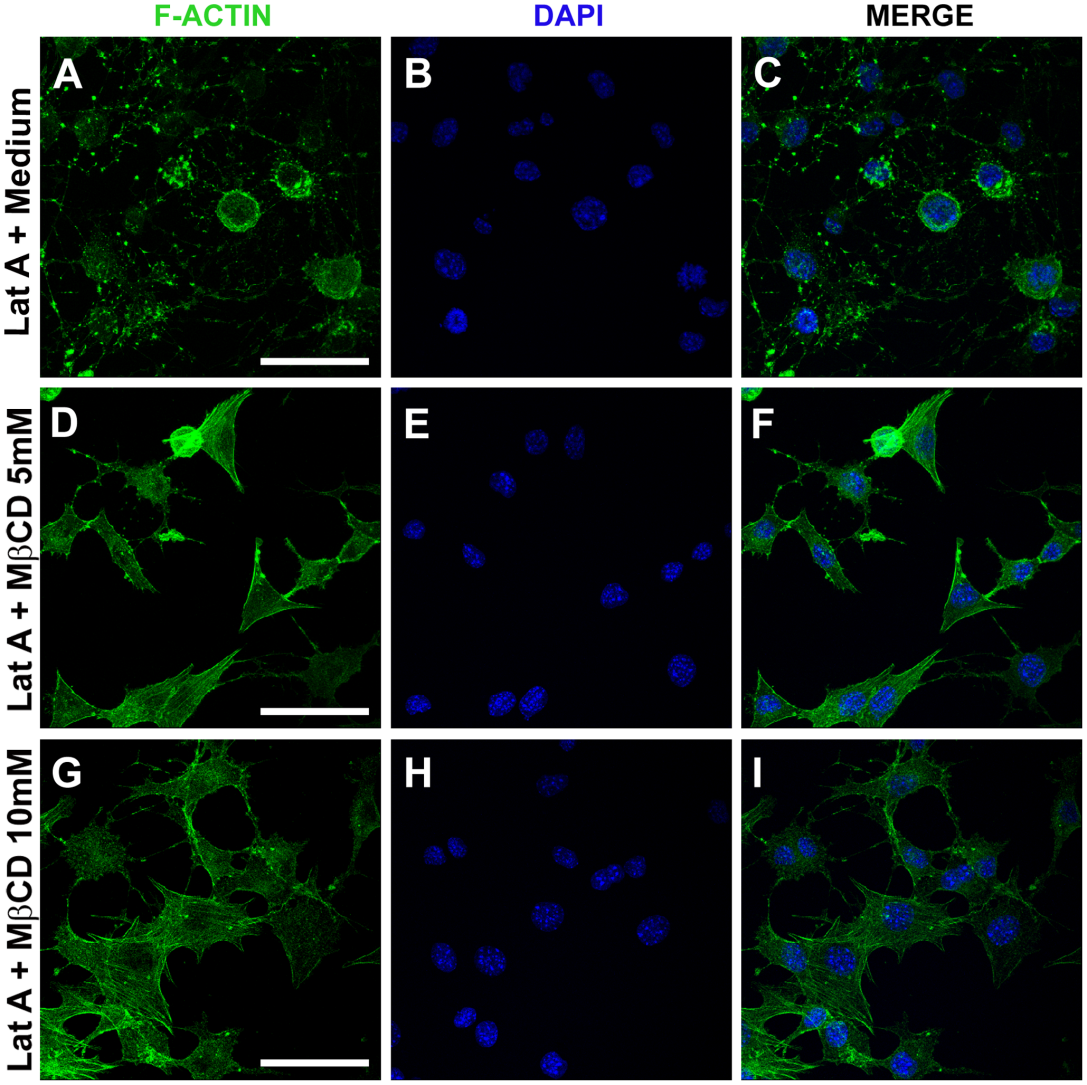
Cholesterol sequestration induces *de novo* actin polymerization. Fibroblasts were submitted to the following treatments: 95 nM of Latrunculin-A (Lat A) for one hour followed by washing with HBSS+ and incubation with fresh DMEM, without serum, for 45 minutes (A–C); previous incubation with Lat-A followed by washing with HBSS+ and incubation with 5 (D–F) or 10 (G–I) mM of MβCD diluted in fresh DMEM, without serum, for 45 minutes. The cells were fixed simultaneously and processed for F-actin cytoskeleton labeling using Phalloidin-FITC. Cell nuclei were stained with DAPI. Three independent experiments were performed in triplicates and at least 50 different cells were observed per group. Each selected image represents the most predominant actin cytoskeleton cell morphology of that experimental condition. Scale bars are all 10 µm.

The results described above confirmed that cholesterol removal changes actin cytoskeleton organization in fibroblasts and activates Rho GTPase, which is known to induce actin polymerization. However, whether the stress fibers induced by cholesterol sequestration resulted from existing F-actin or from *de novo* formation of new F-actin filaments was not known. To examine this issue, we pre-treated cells with 95 nM Latrunculin-A (Lat-A), a drug that disrupts F-actin cytoskeleton by sequestering actin monomers, for 1 hour before treatment with MβCD. As controls, fibroblasts treated with Lat-A were washed and re-incubated in fresh media without the drug for an additional 45 minutes. Figure 3A–C show control cells treated with 95 nM Lat-A for 1 hour followed by 45 minutes of incubation with fresh medium. As expected, these fibroblasts exhibited extensive disruption of F-actin cytoskeleton. Figures 3D–F show cells previously treated with 95 nM Lat-A for 1 hour followed by 45 minutes incubation with 5 mM MβCD. In these cells it was possible to observe a complete reorganization of the F-actin, where most of the cells presented cytoskeleton organization similar to control non-treated cells (Fig. 1A–C). These results demonstrate that cholesterol extraction with MβCD treatment is able to induce *de novo* actin polymerization. Treatment of cells with 10 mM MβCD after 95 nM Lat-A incubation led to a full recovery of the actin cytoskeleton organization, with all cells in the field showing normal distribution of F-actin structures (Fig. 3G–I). These results demonstrate that cholesterol removal not only promotes actin filament reorganization but can also induce its de novo polymerization, which is more prominent in the case when the highest concentration of MβCD was used.

### Cholesterol Removal Alters Membrane-cytoskeleton Mechanical Properties

**Figure 4:**
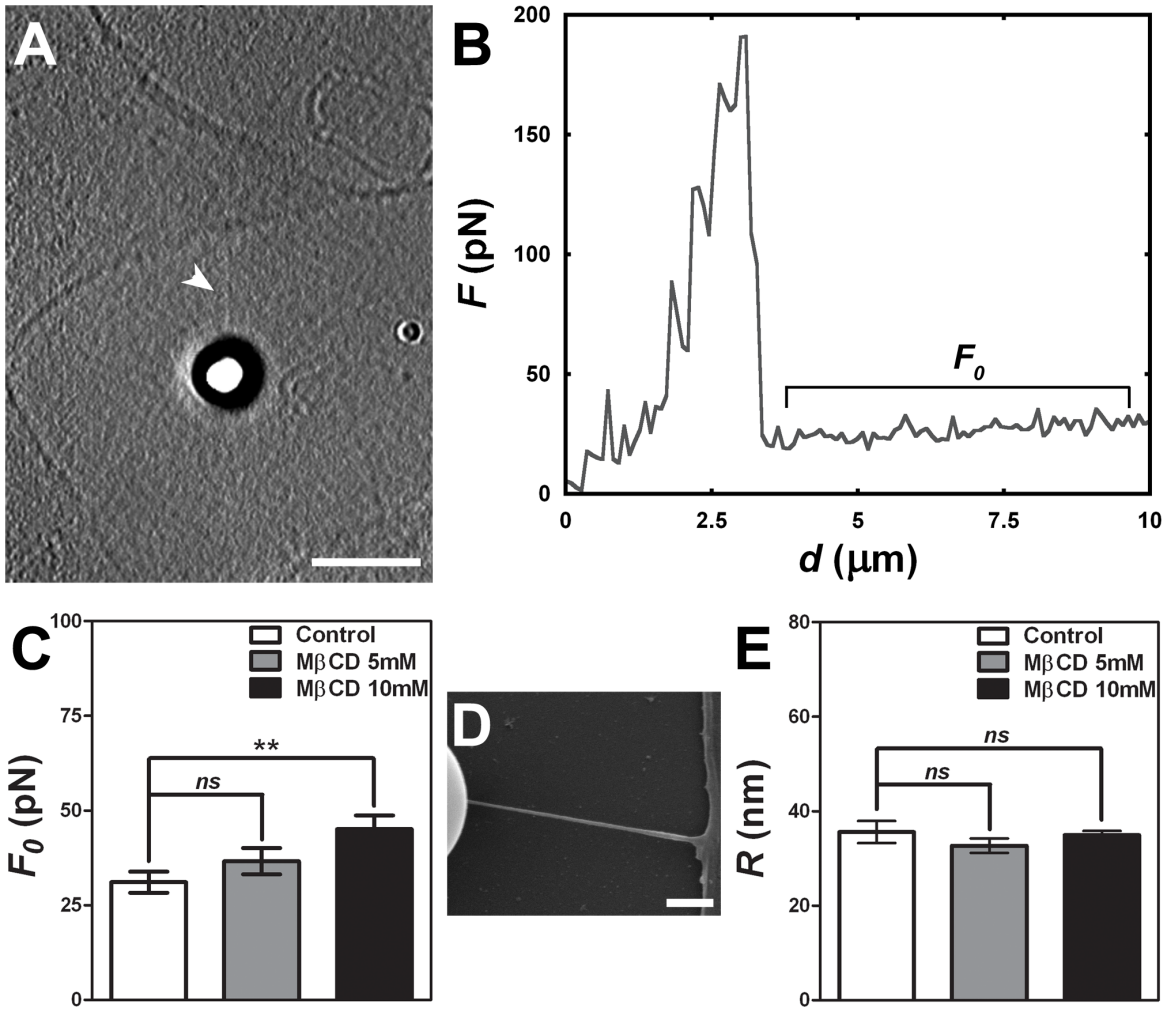
Cholesterol sequestration and tether extraction. (A) Typical image of a tether extracted from a fibroblast, indicated with a white arrowhead. Scale bar is 5 µm. (B) Typical force versus displacement plot showing the plateau corresponding to *F_0_*. (C) Plot representing the mean values of the force *F_0_* for the control, 5 and 10 mM of MβCD. (D) Representative image of a tether obtained by scanning electron microscopy. Scale bar is 1 µm. (E) Plot representing the mean values of the radii obtained for control 5 and 10 mM of MβCD. Three independent experiments were performed and at least 20 different cells were analyzed per each group. The plotted results represent the mean ± standard error of all the measurements. *ns* means no significant difference and ** means p<0.01 in student t-test.

Once we determined that cholesterol removal was able not only to reorganize, but to induce *de novo* formation of F-actin filaments, we proceeded to test the effects of these membrane-cytoskeleton modifications on the mechanical properties of the cells. For this, both control and MβCD treated fibroblasts were submitted to tether extraction using the OT technique. Once the tether is formed (Fig. 4A), the force *F_0_* required to extract it is uniform and appears as a plateau in the force versus displacement curve (Fig. 4B). We measured *F_0_* for control and cholesterol sequestered fibroblasts and the results showed that the lower the cholesterol content in cell membranes, the higher *F_0_* forces were needed to pull the membrane tether (Fig. 4C).

We also measured tethers’ radii (*R)* for each experimental condition by using scanning electron microscopy (Fig. 4D–E) and used both *F_0_* and *R* measurements in equations 3 and 4 to determine the surface tension (*σ)* and the bending modulus (*κ*) of cells, two cellular mechanical properties (Table 2). We observed that cholesterol sequestration, induced by 5 and 10 mM MβCD treatment, significantly increased the surface tension values when compared to untreated cells. Bending modulus was not altered upon treatment of cells with 5 mM of the drug, but an increase could be observed when cells were treated with 10 mM of MβCD when compared to control non-treated cells.

**Table 2:** Cholesterol depletion increased surface tension and bending modulus.

	Surface tension σ(pN/µM)	Bending modulus(pN. µM)
Control	67±9	0,18±0.02
MβCD 5 mM	95±9	0,18±0.02
MβCD 10 mM	102±9	0,25±0.03

These results demonstrate that cholesterol sequestration affects surface tension and bending modulus. Additionally it also indicates that in scenarios where cholesterol is reduced, cells become more rigid.

### Cholesterol Removal changes Membrane-cytoskeleton Dynamics

**Figure 5:**
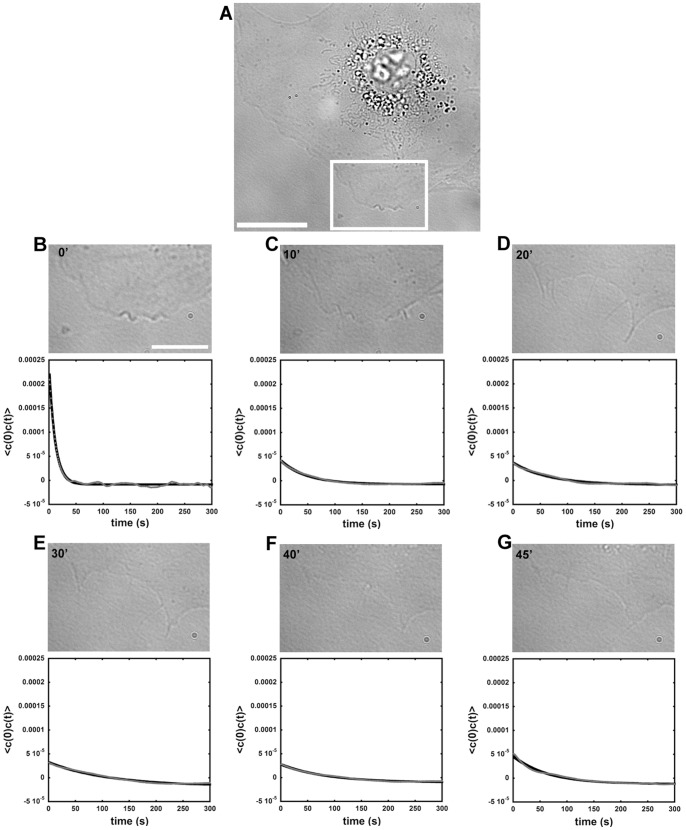
Defocusing microscopy of cells during MβCD treatment. (A) Representative DM image showing a control untreated fibroblast. Scale bar: 20 µM. (B) The region of the inset in (A) shows control case. (C–G) Same region of the inset in (B) at different stages of cyclodextrin treatment. Temporal correlation functions for each corresponding time interval are also displayed under each representative images. Scale bar: 10 µM.

**Figure 6:**
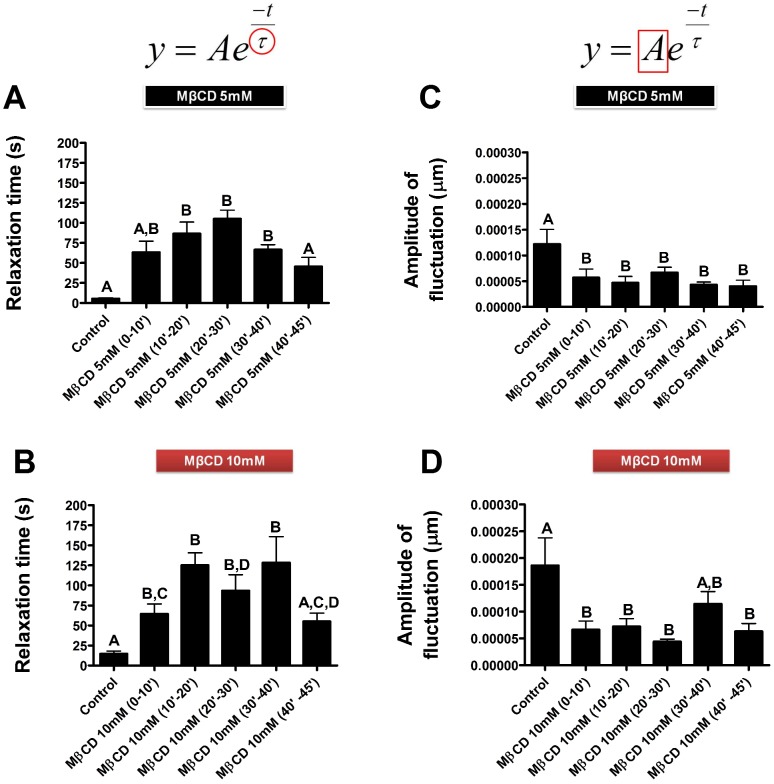
Treatment with MβCD alters relaxation time and amplitude of curvature of cellular membranes. Images of untreated cells were collected during 10 minutes. Following it, cyclodextrin was added and images continued to be collected for the subsequent 45 minutes. Images were analyzed and the relaxation time τ (A and B) and the amplitude of curvature *A* (C and D) of fibroblasts treated either with 5 (A and C) or 10 mM (B and D) of MβCD was obtained according to the equation above graphs. Five independent experiments were performed and at least three regions of each one of the five cells were analyzed. The plotted data represent a mean ± standard error of these five independent experiments. Different letters indicate statistically significant differences (p<0,05 using One-way ANOVA and Newman-Keuls post-test).

The results obtained so far indicated that upon cholesterol extraction actin polymerizes and these membrane-cytoskeleton alterations change the mechanical properties of the fibroblasts, resulting in an increase in cell rigidity. In order to evaluate how mechanical properties of the cytoskeleton-membrane interface were affected by treatment with MβCD we used, for the first time in cholesterol sequestering experiments, a technique called defocusing microscopy (DM). Using this technique we were able to visualize the changes in cellular mechanics along the process of cholesterol sequestration induced by treatment with MβCD. For these experiments, fibroblasts were plated in individual wells and single cell images were taken continuously at 1 frame per second. We analyzed 10 minutes movie cuts from the whole experiment. During the first 10 minutes images were acquired from untreated fibroblasts. After this, MβCD (either at 5 or 10 mM) was added and images continued to be collected for another 45 minutes. Representative frames are shown in Figure 5, showing pre-treatment control images (Fig. 5A and 5B 0′) and images from 10–45 minutes after addition of 10 mM MβCD (Fig. 5C–G). Temporal correlation fits are shown below each of the frames (Fig. 5B–G). The data was fitted using a temporal autocorrelation function [29], [32] so that the values of the relaxation time (*τ*) and amplitude of curvature (*A*) could be determined (Fig. 6). Analysis of the relaxation time observed during cholesterol sequestration with 5 mM of MβCD showed a more defined behavior when compared to the data obtained from cells treated with 10 mM MβCD. Nonetheless, in both cases it was possible to observe that the relaxation time increased after MβCD addition and then decayed during the final 5 minutes of the experiment. The highest relaxation time values observed after treatment with 5 mM MβCD occurred between 10–30 minutes after addition of the drug, whereas for cells treated with 10 mM MβCD the highest values occurred 10–40 minutes after treatment (Fig. 6A and B). Additionally, we also analyzed the amplitudes of the exponential decay curves. According to DM theory, these amplitudes indicate variations in cell curvature [29]. Both treatments, with 5 and 10 mM MβCD, exhibited a reduction in the amplitude of membrane-cytoskeleton fluctuation, which occurred immediately after the addition of the drug and continued to the subsequent times (Fig. 6C and D). In agreement with the results obtained from OT, these experiments showed a clear alteration in cellular mechanical properties, indicating that after cholesterol extraction cells became stiffer when compared to control conditions. Since we were able to test this parameter along the treatment with the drug we were able to show that the process is fast and happens in the first ten minutes of exposure to the drug.

### MβCD Treatment alone Triggers Lysosomal Exocytosis, Independently of the Calcium Sensor Protein Synaptotagmin VII

**Figure 7:**
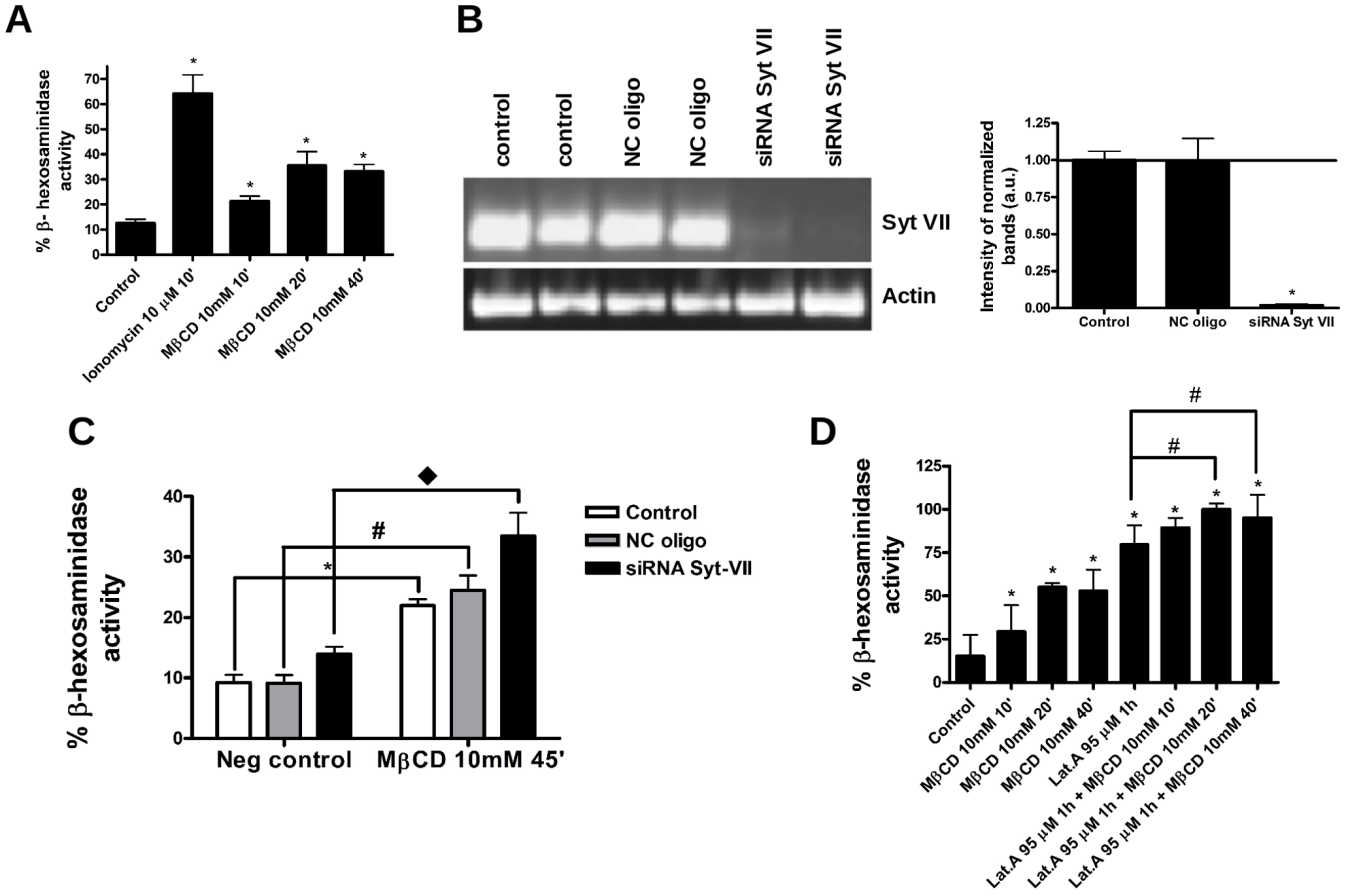
Lysosome exocytosis due to cholesterol removal is syt-VII independent and mobilizes a specific lysosome pool. (A) Lysosomal exocytosis assay of fibroblasts exposed to 10 mM of MβCD for 10, 20 or 40 minutes. Ionomycin was used as a positive control for lysosomal exocytosis. Asterisks (*) indicate statistically significant differences between control and treated groups (p<0.05 using student t-test). (B) Knocking down of syt-VII in NRK cells was confirmed by RT-PCR, using sytVII or actin specific primers. Left: Control non-transfected NRK (control), NRK transfected with medium GC siRNA – negative control oligo (NC oligo) and NRK transfected with syt-VII siRNA oligo (siRNA sytVII). Right: quantification of the intensity of the gel bands (C) Lysosomal exocytosis assay of non-transfected NRK (control cells), NRK transfected with medium GC siRNA – negative control oligo (NC oligo) and NRK transfected with syt-VII siRNA oligo (siRNA sytVII). Asterisk (*), hashtag (#) and diamond (υ) indicate statistically significant differences between MβCD treated groups and their respective controls (p<0.05 using student t-test). (D) Lysosomal exocytosis assay of fibroblasts pre-treated or not with Lat-A 95 nM, for one hour, followed or not by incubation with MβCD 10 mM for 10, 20 or 40 minutes. Asterisks (*) and hashtags (#) indicate statistically significant differences between control and MβCD treated groups (*) or Lat-A and Lat-A+MβCD treatment (#) (p<0.05 using student t-test). (A, C and D) The results are expressed as the ratio between β-hex activity in the cell supernatant and total β-hex activity and are shown as mean of triplicates ± standard error. Three independent experiments were performed in triplicates. Pooled data are shown as mean of all results ± standard error.

As we and others recently reported, cholesterol sequestration with β-cyclodextrins treatment induces lysosomal exocytosis in fibroblasts, cardiomyocytes and epithelial cells [13], [14], [15]. However, the need of intracellular calcium signaling and/or extra stimuli for MβCD induced exocytic events has remained unclear. By using wild type BALB/c and NPC1 mice, Chen *et al.* (2010) reported that 2-hydroxypropyl-beta-cyclodextrin (HPβC) alone was capable of inducing lysosomal exocytosis without the concomitant use of calcium ionophores. However, this study also proposed that, for the cells analyzed, these exocytic events were dependent on extracellular calcium [13]. Xu *et al.* (2012), on the other hand, reported no effect of cyclodextrin treatment alone in inducing lysosomal exocytosis in MDCK epithelial cells [14]. In our experience with cardiomyocytes, treatment with MβCD alone was sufficient to induce exocytosis of lysosomes, and the process was independent of extracellular calcium [15]. In order to determine whether in fibroblasts MβCD treatment alone was capable of triggering lysosome exocytic events without the participation of calcium, we measured the secretion of the lysosomal enzyme β-hexosaminidase (β-hex). As a positive control we used ionomycin, a calcium ionophore, which is known to induce lysosomal secretion. The results are shown in Fig. 7A. As expected, treatment with ionomycin led to an increase in β-hex activity in the supernanatant of control, untreated cells. Notably, 10, 20 and 40 minutes of incubation with MβCD also induced enhancement in β-hex exocytosis in relation to untreated cells. These results demonstrate that cholesterol sequestration by MβCD is sufficient to induce lysosomal exocytosis in fibroblasts, as previously observed for cardiomyocytes [15]. Since cell viability was not altered by MβCD treatment (data not shown), we also did not expect membrane injuries in this condition, which would lead to extracellular calcium influx, suggesting that lysosome exocytosis induced by cholesterol removal was also independent of intracellular calcium increase.

In cardiomyocytes, we previously observed that cholesterol sequestration triggered the exocytosis of peripheral lysosomes, suggesting that there might be two functionally independent sets of these organelles [15]. It had also been shown in the literature that peripherally-docked lysosomes represent the population that fuses with the plasma membrane upon intracellular calcium elevation, under the regulatory effect of synaptotagmin VII (syt-VII) [33], [34]. Syt-VII is a calcium sensor protein present in lysosomes, which is able to detect calcium in the intracellular milieu and control the triggering and secretion of those organelles [12]. Based on these data, we decided to investigate whether peripheral lysosome secretion induced by MβCD treatment required Syt-VII. In order to deplete cells in Syt-VII, siRNA knock-down experiments were performed using NRK cells. Cells were either transfected with an oligo directed to Syt-VII (siRNA SytVII - Syt7RSS339874– Invitrogen, Carlsbad, CA, USA) or a negative control siRNA (medium GC oligo), as an experimental control. After confirming the success of the Syt-VII knockdown by RT-PCR (Fig. 7B) we performed a lysosomal exocytosis assay in control or MβCD treated cells (Fig. 7C). We found that the absence of Syt-VII did not abolish lysosomal exocytic events triggered by MβCD treatment, demonstrating that the exocytosis of peripheral lysosomes induced by cholesterol sequestration does not require the calcium sensor Syt-VII (Fig. 7C), and therefore is independent of intracellular calcium signaling. We have also shown that MβCD induced lysosomal exocytosis in primary cardiomyocyte (CM) cultures was independent of calcium by performing the β-hex assay in CM cultures treated with 10 mM of MβCD in the presence of 1 mM of BAPTA-AM, an intracellular calcium chelating agent (Fig. S1).

### Lysosomal Exocytosis Triggered by MβCD may be Controlled by Actin Polymerization

There is significant evidence in the literature that the actin cytoskeleton plays a role in exocytic events. While some authors demonstrated that the actin cortical cytoskeleton works as a barrier to vesicle secretion [35], [36], [37], others have shown that actin can act as a facilitator of exocytic events through the formation of a readily releasable pool of vesicles, near the plasma membrane and/or by controlling vesicle fusion of pre-docked vesicles [38], [39].

Since cholesterol removal in our assays led to an increase in lysosomal exocytosis, without the apparent participation of calcium, we set out to investigate whether actin polymerization and stress fiber formation induced by cholesterol sequestration could be playing a role in this process. In order to answer this question, fibroblasts were pre-treated or not with Lat-A and submitted to incubation with 10 mM of MβCD for 10, 20 or 40 minutes. β-hex activity was measured for each condition, including control cells and cells treated only with Lat-A (Fig. 7D). Notably, cells treated only with Lat-A exhibited a considerable level of β-hex release when compared with control cells (Fig. 7B). Moreover, Lat-A incubation and subsequent 10 mM MβCD treatments, for 20 and 40 minutes, induced higher levels of lysosomal exocytosis when compared to the exocytosis in cells only exposed to Lat-A (Fig. 7D).

**Figure 8:**
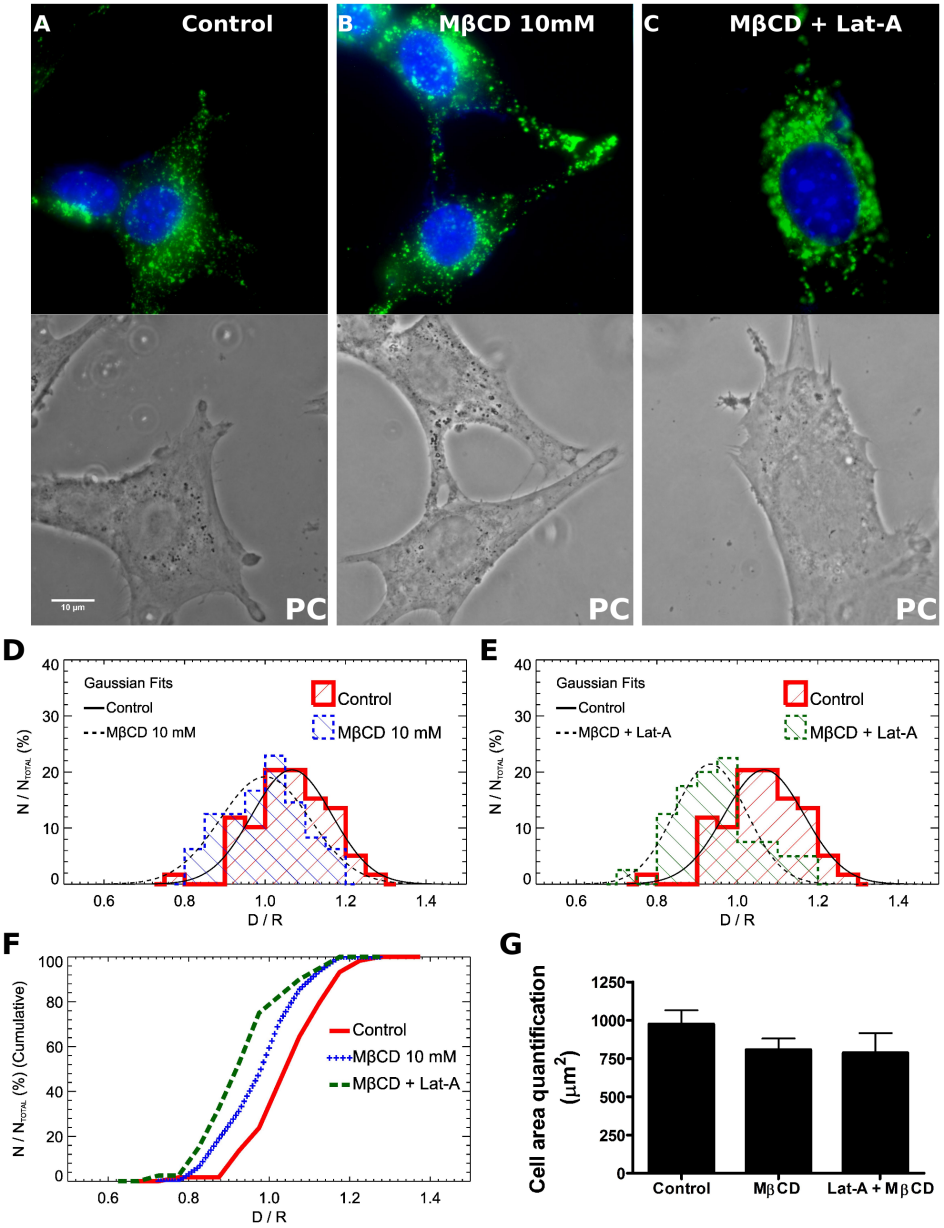
Cholesterol sequestration mobilizes a different lysosomal pool in comparison to cytoskeleton disruption. (A–C) Representative images of control, MβCD 10 mM treated and Lat-A+MβCD 10 mM treated cells, fixed and immunolabeled for DAPI and LAMP-1. PC: phase contrast. Scale bar: 10 µm. (D–E) Quantitative analysis of lysosomal distribution, relative to cell nuclei, in MβCD 10 mM (D) and Lat-A 95 nM+MβCD 10 mM (E) in comparison to control non-treated cells. The mean distance between a lysosome and its respective cell center is represented by letter *D* and the mean lysosome distance relative to the mean nucleus’ radius *R* was defined as the ratio *D/R*. The histogram for the control (non-treated) lysosomal distribution is represented with a red solid thick line, MβCD 10 mM is represented with a blue dashed line and Lat-A+MβCD combined treatment is represented with a green dashed line. Overlaid Gaussian fits to each distribution were added in order to highlight the main statistical trend of each diagram. The x-axis of each graph represents the ratio *D/R* whereas the y-axis represents the percentage of analyzed nuclei from each treatment normalized by total cell number. (F) Cumulative frequency of the histograms. Plus sign blue line represents MβCD 10 mM treated cells, dashed green line represent Lat-A+MβCD 10 mM treated cells and red continuous line represent control untreated cells. The Kolmogorov- Smirnov (KS) statistical test was performed to compare the cumulative frequency distributions. Statistically significant differences were obtained for MβCD 10 mM 0.25% (p = 0.0025), Lat-A+MβCD 10 mM 0.000091% (p = 0.00000091) treated cells in relation to control, and for MβCD 10 mM in relation to Lat-A+MβCD 10 mM 4,3% (p = 0.043). (G) Cell area comparison between control and treated groups. No statistical differences were detected among them (p>0.05 student T-test). Three independent experiments were performed in triplicates and at least 20 cells from each condition were analyzed. Data are shown as mean of at least 20 cells from a representative experiment ± standard error.

We also performed qualitative and quantitative analysis of lysosomal distribution in control and MβCD treated cells previously exposed or not to Lat-A (Fig. 8). Fibroblasts treated only with Lat-A were not analyzed since these cells present a great variation in their morphology and cell area in comparison to control and the other treatments (see Fig. 3C). Figure 8A–C shows representative images of lysosomal distribution by immunofluorescence in control fibroblasts (Fig. 8A), fibroblasts treated with 10 mM MβCD alone (Fig. 8B) or after Lat-A 95 nM incubation (Fig. 8C). Qualitatively, we can notice that fibroblasts with less cholesterol content in their membranes (Fig. 8B) exhibit, in average, lysosomes nearer their respective nuclei in comparison to untreated cells (Fig. 8A). Interestingly, fibroblasts treated first with Lat-A and then with MβCD 10 mM (Fig. 8C) showed lysosomes even closer to cell nuclei in comparison to cells treated only with MβCD (Fig. 8B). In order to precisely determine these differences, the same images were used to perform a quantitative assay of lysosomal dispersion (Fig. 8D and E). First, for each isolated nucleus it was calculated the mean radius (R). The next step was to select each lysosome associated with its respective nucleus and to measure the mean distance between a lysosome and cell center (D). Lysosomes farther than 1.5 radii were not considered in the calculation to avoid inclusion of lysosomes from neighboring cells. Finally, the mean lysosome distance (D) relative to the mean nucleus’ radius (R) was defined as the ratio D/R, where values closer to one indicate lysosomes are nearer the perinuclear region whereas the opposite indicates lysosomes are more frequent at cell borders. This ratio D/R was measured for several groups of lysosomes associated with each nucleus in the different treatments. The results of this analysis are distributions of D/R values associated to each drug treatment, and are represented as histograms (Fig. 8D and E). Gaussian fits from control cells show that the majority of lysosomes are preferentially localized at ratio 1.07 from cell center whereas the peak of Gaussian fits from 10 mM MβCD treated cells show a ratio of 1.00 (Fig. 8D). Gaussian distribution from fibroblasts treated with Lat-A and MβCD indicates that lysosomes are preferentially located at ratio 0.93 (Fig. 8E). Cumulative frequencies of lysosomes (Fig. 8F) from the histograms of figures 8D and E were plotted and analyzed using Kolmogorov-Smirnov statistical test. Statistically significant differences were observed among all groups. In order to prove that lysosomal dispersion had not been altered due to changes in cell area, we also quantified the mean cell surface area for the different conditions and no statistically significant differences was detected among groups (Fig. 8G).

The results obtained for lysosomal secretion and distribution upon treatment with each drug, Lat A and MβCD, alone or in combination, strongly suggest that fibroblasts have at least two distinct pools of lysosomes, which respond differently to exocytosis stimulus triggered by cholesterol sequestration or by actin cytoskeleton disruption.

## Discussion

In this study we investigated the relationship between two cellular events induced by membrane cholesterol sequestration: lysosomal exocytosis and reorganization of the actin cytoskeleton. We found that actin reorganization and polymerization influences the unregulated, calcium-independent lysosomal secretion induced by MβCD treatment.

It is well known that the plasma membrane and actin cytoskeleton are intimately connected, and that their association may occur in membrane microdomains enriched in cholesterol and sphingolipids, known as lipid rafts [31]. Cytoskeleton-associated or cytoskeleton proteins such as actin, tubulin, myosin, α-actinin and supervillin form clusters and bind to membrane microdomains [17], [40]. Membrane rafts also concentrate certain integrins which regulate cytoskeleton organization, membrane trafficking and connections with the extracellular matrix through focal adhesion points [41]. Additionally, it has also been shown that cholesterol extraction by cyclodextrin treatment alters the actin cytoskeleton organization with the formation of stress fibers [21]. In this latter study, performed with immortalized osteoblasts, the authors showed that serum deprivation followed by 60 minutes cholesterol sequestration with 5 mM MβCD caused stress fiber formation through Rho activation [21]. In agreement with this study, using a mouse fibroblast cell line we also found that cholesterol removal from plasma membrane induces actin rearrangements as well as Rho activation. However, we observed that cholesterol removal itself, in the absence of previous serum deprivation, was sufficient to produce these effects. Most importantly, we have shown that actin remodeling upon MβCD treatment occurs not exclusively due to F-actin rearrangement, but through *de novo* polymerization of actin filaments. Treatment with MβCD after treatment with Latrunculin A, a drug that depolymerizes the actin cytoskeleton, was capable to restore cellular actin filaments.

We found that these changes in the actin cytoskeleton reflected on the cellular mechanical properties, with MβCD treated cells becoming stiffer when compared to control non-treated fibroblasts. In fact, it was previously demonstrated that both surface tension σ and bending modulus κ can considerably vary not only between different cell types but also among the same cells that are performing different functions [26]. Under all these conditions, changes in the cytoskeleton, as well as in its interaction with the plasma membrane can support these results. This seems also to be the case for our findings in this paper. Tether extraction experiments using optical tweezers in control and cholesterol sequestered fibroblasts clearly showed that the surface tension σ, increases significantly in cells treated with the cyclodextrin. Moreover, we used for the first time the defocusing microscopy technique to study time-dependent effects of cholesterol removal from cells. We showed that the relaxation time τ significantly increases in the beginning of MβCD treatment and then returns to lower values, more similar to controls, at the end of the treatment. The increase in relaxation time at the beginning of cholesterol sequestration may be explained by an enhancement in cell viscosity due to the cytoskeleton rearrangement that occurs during that period. With this methodology we were also able to obtain information on membrane fluctuation amplitude, and we showed that during cholesterol removal the amplitude decreased, indicating that membrane-cytoskeleton fluctuation become less prominent upon cholesterol sequestration. This time-dependent analysis of cholesterol removal provided by DM is a very important result since we showed, through this technique, for the first time, the kinetic behavior of cholesterol extraction from cell plasma membrane. Using this technique we showed that the effect of cholesterol removal on cellular mechanics occurs very fast, in the first 10 minutes of exposure to the drug. Also, the bending modulus values increased when cells had less amount of cholesterol in their membranes. Bending modulus can also be evaluated indirectly by fluctuation amplitude obtained by DM since both are inversely proportional quantities. Since we saw a decrease in cell curvature amplitude in DM results it was plausible to conclude that an increase in bending modulus could be observed. Additionally, contrary to surface tension, which is constant and uniform along the entire plasma membrane [42], bending modulus can be adjustable by other mechanisms and is important for dynamic cell remodeling and movement [43]. Although lipid composition could also account for alterations in the mechanical properties of cells, the changes observed upon cholesterol removal seem to be mainly related to the actin cytoskeleton polymerization and rearrangement. In previous works with isolated model membranes, it was demonstrated that the elastic area compressibility modulus, which is defined as the resistance of the membrane to isotropic area dilatation, increased after cholesterol addition [44], [45]. Moreover, membrane tension at lysis (tension measured at the moment that a vesicle ruptures after being aspirated by a micropipette) also increased when cholesterol was added. Thus, the increment in model membrane stiffness was, in these isolated model membranes, due to cholesterol addition and not due to its removal. Moreover it was previously demonstrated that actin microfilament disruption caused by cytochalasin D is able to generate a large decrease in surface tension but a large increase in bending modulus, when compared to control conditions [27]. Thus, it is plausible to assume that an increase in actin polymerization should change the membrane mechanical properties in an opposite fashion to what occurs after cytochalasin D treatment. In fact, our results demonstrate an increase in surface tension and bending modulus after MβCD as expected. Nonetheless, we also need to take into account that the existing theories of tether extraction, which we used to determine the mechanical properties, do not take into account the presence and interaction of the cytoskeleton within the tethers, as demonstrated previously [27]. So the final numerical results obtained are subject to some uncertainties.

Our results are in accordance with previously published studies that used different methodologies, such as micropipette aspiration and atomic force microscopy. These studies showed that reduction in cholesterol content leads to an increase in cell rigidity [19], [20], [46]. Khatibzadeh and collaborators (2012) also showed that cholesterol sequestration in HEK 293 cells increases the tether force values as well as the rigidity and adhesion energy of those tethers [47]. Although they also used tether extraction by OT, tether radius was measured by treatment of images obtained from an optical microscope. However, Pontes and collaborators demonstrated that SEM is a much better method to measure tether radius, since the values obtained are smaller than the resolving power limit of an optical microscope [26], [27]. In our experiments we used SEM and followed the procedures described before by Pontes et al. (2011 and 2013) [26], [27]. Despite this difference, the conclusions presented by Khatibzadeh and collaborators (2012) are also in agreement with our results. The increase in cell rigidity probably results from the fact that before cholesterol removal, membrane rafts are clustered and organized in specific regions of plasma membrane, where specific transmembrane proteins responsible for the connection between cytoskeleton and extracellular matrix reside. After cholesterol removal, the rafts are disrupted and these proteins become spread over the entire plasma membrane, establishing connections between membrane and cytoskeleton along the entire cellular surface. Protein dispersion throughout the membrane upon cholesterol removal has been shown in other studies [48], [49]. This hypothesis is also consistent with data shown in the study by Norman and collaborators which demonstrated that cholesterol sequestration enhances focal adhesion points [50].

Variations in surface tension can also be observed when cells are exposed to hypotonic solutions, due to significant variation in cell volume. Thus, it has long been discussed that in order to protect cells against lysis, intracellular vesicles fuse donating membrane to cell surface [51]. It was shown that during cell spreading, and more recently also during phagocytosis [52], cell area increases over time until plasma membrane reservoir becomes completely sequestered, which also leads to an increase in membrane tension. Tension on the membrane is then compensated by the exocytosis of a vesicle pool [53], [54]. Altogether, these findings put membrane tension as a very important regulator of some biological processes. Considering this scenario and the fact that cholesterol removal enhances cell surface tension, we should expect that in this condition we would also observe exocytosis of an intracellular membrane reservoir. In fact, previous studies have shown that cholesterol sequestration through cyclodextrin treatment alters the regulation of several exocytic events, such as the release of synaptic vesicles in the peripheral and central nervous system or sperm acrosome and insulin secretion [8], [9], [55], [56], [57]. Recently, our group, as well as Chen and co-workers, have also shown that membrane cholesterol sequestration leads to lysosomal exocytosis in cardiomyocytes and fibroblasts [13], [15]. Here we showed that lysosomal exocytosis occurs when cholesterol is removed from a fibroblast cell line, concomitantly with the increase in surface cell tension, measured by OT. Therefore, lysosome secretion may be a reaction triggered by the need to restore basal surface tension values. However the exact mechanism by which cholesterol sequestration and surface tension induces these exocytic events were still not clear.

Membrane fusion events, such as synaptic vesicle and lysosomal exocytosis as well as other types of vesicle secretion, are usually regulated by calcium and occur through a mechanism dependent on proteins from the SNARE complex [58], [59], [60]. Some of these proteins are known to be partitioned in cholesterol-dependent clusters, such as membrane rafts, at which sites vesicles fuse [61], [62]. It is possible that raft disorganization could change the distribution and/or function of SNARE proteins, disturbing the exocytic events regulated by these proteins. In fact, SNARE redistribution upon cholesterol sequestration was reported for sperm cells and PC12 cells. In the case of PC12 cells, it was shown that SNARE localization in rafts act as negative regulators of synaptic vesicle secretion, and reducing SNAP 23 partitioning to raft sites enhanced vesicle exocytosis [63]. Interestingly, SNAP 23 is one of the SNARE complex proteins involved in lysosomal fusion events [60]. Therefore, SNARE re-localization could be a possible explanation for lysosome exocytosis triggered upon MβCD treatment. However, it is well accepted that, in regulated exocytosis, calcium is important for the final fusion event, especially for docked/primed vesicles. Even though SNAREs are most likely already partially zippered in this state, full zippering is believed to occur only when calcium is present (reviewed by Jahn and Fasshauer, 2012) [64]. Calcium binding to synaptotagmin would trigger fusion either by activating SNAREs or by lowering the activation energy barrier for fusion [64]. In our previous work with cardiomyocytes we have demonstrated that cholesterol sequestration leads to lysosomal exocytosis independently of extracellular calcium [15]. Here we show that this cholesterol-induced lysosomal exocytosis in cardiomyocytes is also independent of intracellular calcium (Fig. S1). We also show that lysosomal secretion triggered by MβCD treatment of fibroblasts was independent of Syt-VII, the calcium sensor in lysosomes [12]. Chen *et al.* (2010), using wild type BALB/c and NPC1 mice (a experimental model for Niemann Pick Type C disease which is characterized by cholesterol accumulation in the endosomal/lysosomal system), reported that 2-hydroxypropyl-beta-cyclodextrin (HPβC) alone was also able to induce lysosomal exocytosis without the concomitant use of calcium ionophores. However, this study also proposed that, for the cells analyzed, these exocytic events were dependent on extracellular calcium [13]. This apparent discrepancy in relation to our previous published data is probably due to the pair cyclodextrin-cell utilized. Also, since MβCD presents more affinity for cholesterol then HPβC [65], the lysosomal exocytosis triggered by MβCD possibly shows a different pattern in relation to the exocytosis provoked by HPβC incubation. Xu *et al.* (2012), on the other hand, reported no effect of cyclodextrin treatment alone in inducing lysosomal exocytosis in MDCK epithelial cells [14]. This was probably due to the fact that after inducing polarization of those cells by using transwell assays, the lysosomal secretion occurred more prominently in specific cell regions. Considering that exocytosis triggered by cholesterol sequestration is refractory to calcium in our model, it remained to be determined if some other factor was also contributing to this event.

While some authors demonstrated that the actin cortical cytoskeleton works as a barrier to vesicle secretion [35], [36], [37], there are increasing evidence in the literature indicating that actin can also act as a facilitator of exocytic events through the formation of a readily releasable pool of vesicles, near the plasma membrane and/or by controlling fusion of these pre-docked vesicles [38], [39], [66], [67]. In our previous work with cardiomyocytes, we showed that cholesterol sequestration triggered the exocytosis of lysosomes preferentially located at the cell cortex [15], which are the ones that are usually regulated by calcium and Syt-VII [33]. We also demonstrated in this work that fibroblasts treated with MβCD exhibit the same behavior observed for cardiomyocytes when cholesterol was sequestered from those cells. With the goal of correlating these data with our new results demonstrating an increase in cell tension and actin polymerization due to cholesterol sequestration, we investigated the role played by actin cytoskeleton in lysosomal exocytosis triggered by cholesterol extraction. Our results show that, as observed for MβCD treatment, Lat-A treatment alone induced exocytosis of lysosomes. Latrunculin-A -induced vesicle secretion was also reported in hippocampal neurons [68]. In this case, actin and/or actin-associated proteins were proposed to restrict vesicles from becoming primed for fusion. Therefore, in this scenario, actin disruption, by Lat-A treatment, may allow the exocytosis of a more internally located pool of vesicles. In fact, it has been shown for chromaffin cells that there are two distinct vesicle pools: one located near the plasma membrane which behaves as a readily releasable pool and does not suffer any influence from cytoskeleton (but could be linked to the actin cytoskeleton trough accessory proteins) and a second pool of granules, in the inner cytoplasm, which is intimately connected to actin microfilaments [37], [69]. Treatment with actin disrupting drugs such as cytochalasin-D was shown to induce the exocytosis of this inner pool, while the docked pool was not secreted due to cytoskeleton disruption [35]. We also demonstrated for fibroblasts that cytoskeleton disruption promoted by Lat-A followed by cholesterol removal with MβCD led to a secretion of a more internal pool of lysosomes corroborating data obtained for chromaffin cells. Since lysosomes recruited for fusion by cholesterol sequestration appear to be the ones located at cell periphery, and most likely the ones that are already docked, they are probably distinct from the pool recruited by Lat-A treatment. In fact, lysosome exocytosis triggered by MβCD after Lat-A incubation showed exocytic levels higher than the ones observed for each drug alone, showing that the effects of the two drugs might be additive. These results suggest the existence of two distinct pools of lysosomes that are regulated differently. In this case, Lat-A incubation would lead to the release of a more internal pool of lysosomes, while the following treatment with MβCD, which we have proved to restore cortical actin filaments, would promote the exocytosis of the peripheral pre-docked ones. Our lysosomal dispersion analysis also corroborate this hypothesis, since it shows that cells pre-treated with Lat-A, before exposure to MβCD, present lysosomes more restricted to the cell nuclei when compared to cells treated with MβCD alone. As mentioned before, extensive evidence in the literature has implicated actin and its molecular motors as important players in exocytic events [39], [70], [71], [72], [73]. Some of these authors have shown that Rho activation is important for actin participation in exocytosis [39], [70], [71]. We have shown that MβCD treatment and cholesterol sequestration also induce Rho activation. Thereby, we suggest that cholesterol sequestration induces exocytosis of the external pool of lysosomes by reorganizing the actin cortical cytoskeleton, which would pull this lysosomal pool closer to the plasma membrane, activating components of the exocytic machinery and inducing their exocytosis.

## Supporting Information

Figure S1 **Cholesterol sequestration induces lysosomal exocytosis even in the absence of intracellular Ca^2+^.** Cardiomyocytes were exposed to 10 mM of MβCD, for 10, 20 or 40 minutes, at 37°C, in the presence or absence of 1 mM of BAPTA-AM **(**1,2-Bis(2-aminophenoxy)ethane-N,N,N′,N′-tetraacetic acid tetrakis(acetoxymethyl ester), which is a drug capable of chelating intracellular calcium. For the exocytosis assay, we incubated both supernatant and cell lysate with β-hex substrate. Results are represented as the ratio between β-hex activity in the cell supernatant/β-hex activity in the cell supernatant+β-hex activity in the cell lysate. Data is shown as average of triplicates ± standard error. Equal letters represent statistically equal groups. (p<0.05, one-way ANOVA plus Neuman Keuls).
(TIFF)
