# Supplementary figures and images for: Membrane Cholesterol Removal Changes Mechanical Properties of Cells and Induces Secretion of a Specific Pool of Lysosomes
Source: PLoS One. 2013 Dec 20;8(12):e82988. doi: 10.1371/journal.pone.0082988 (PMC3869752; doi:10.1371/journal.pone.0082988)

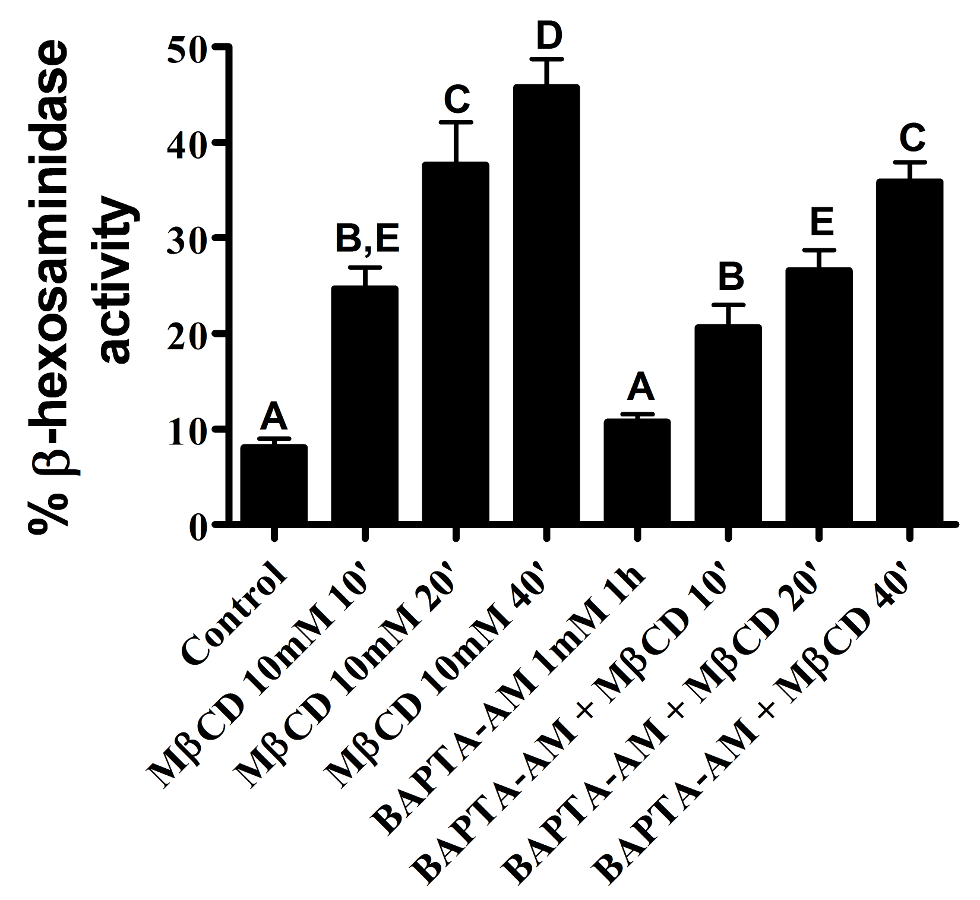

Supplement: Figure S1 — Cholesterol sequestration induces lysosomal exocytosis even in the absence of intracellular Ca2+. Cardiomyocytes were exposed to 10 mM of MβCD, for 10, 20 or 40 minutes, at 37°C, in the presence or absence of 1 mM of BAPTA-AM (1,2-Bis(2-aminophenoxy)ethane-N,N,N′,N′-tetraacetic acid tetrakis(acetoxymethyl ester), which is a drug capable of chelating intracellular calcium. For the exocytosis assay, we incubated both supernatant and cell lysate with β-hex substrate. Results are represented as the ratio between β-hex activity in the cell supernatant/β-hex activity in the cell supernatant+β-hex activity in the cell lysate. Data is shown as average of triplicates ± standard error. Equal letters represent statistically equal groups. (p<0.05, one-way ANOVA plus Neuman Keuls). (TIFF) [file pone.0082988.s001.tiff]
